# Increasing the Scalability of Toxin–Intein Orthogonal Combinations

**DOI:** 10.1021/acssynbio.2c00477

**Published:** 2023-01-27

**Authors:** Rocío López-Igual, Pedro Dorado-Morales, Didier Mazel

**Affiliations:** † Institut Pasteur, Université de Paris, Unité Plasticité du Génome Bactérien, et CNRS, UMR3525, 28 Rue du Dr Roux, F-75015 Paris, France; ‡ Instituto de Bioquímica Vegetal y Fotosíntesis, CSIC and Universidad de Sevilla, Américo Vespucio 40, E-41092 Seville, Spain

**Keywords:** toxin−antitoxin systems, inteins, protein splicing, bacterial killing, microbial synthetic biology

## Abstract

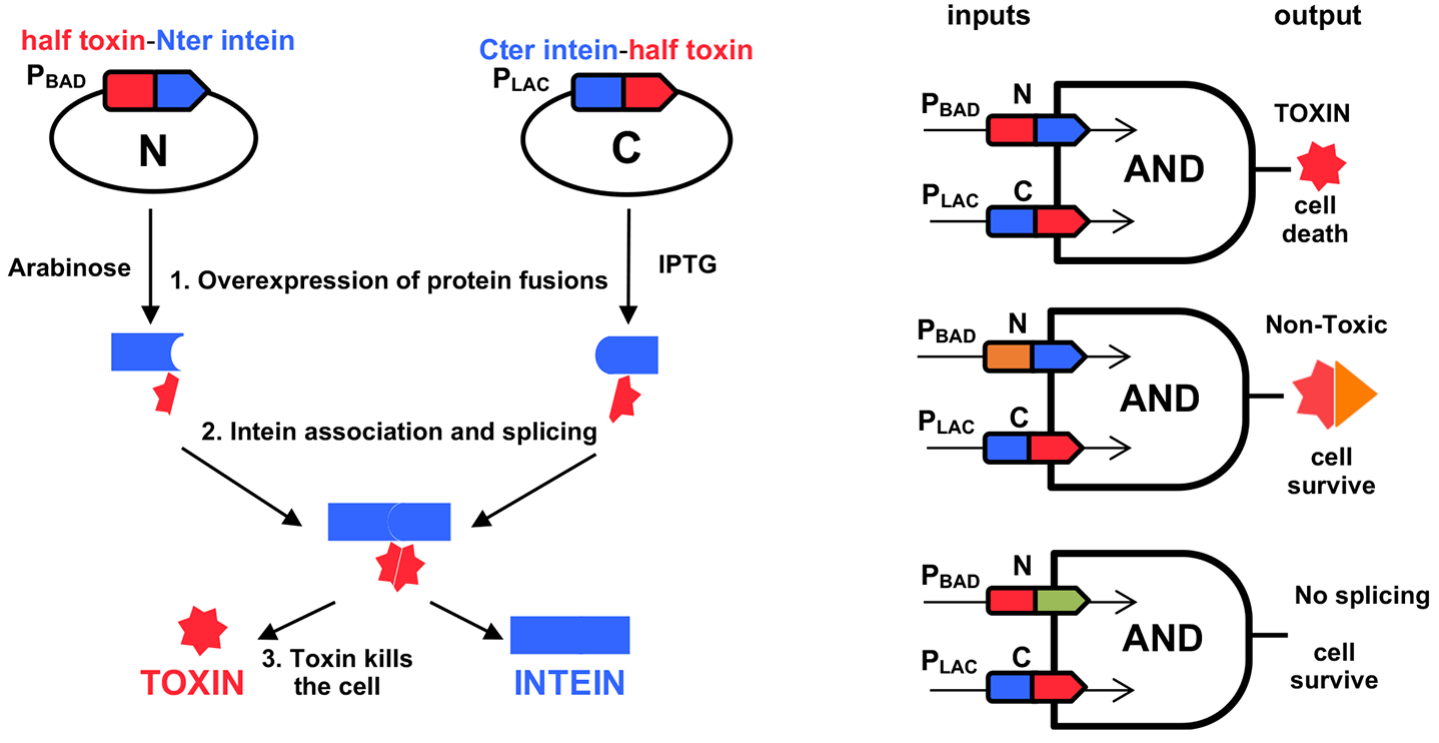

Inteins are proteins embedded into host proteins from which they are excised in an autocatalytic reaction. Specifically, split inteins are separated into two independent fragments that reconstitute the host protein during the catalytic process. We recently developed a novel strategy for the specific killing of pathogenic and antibiotic resistant bacteria based on toxin–intein combinations. Bacterial type II toxin–antitoxin systems are protein modules in which the toxin can provoke cell death whereas the antitoxin inhibits toxin activity. Although our previous system was based on a split intein (iDnaE) and the CcdB toxin, we demonstrated that iDnaE is able to reconstitute four different toxins. To expand the applicability of our system by widening the repertoire of toxin–intein combinations for complex set-ups, we introduced a second intein, iDnaX, which was artificially split. We demonstrate that iDnaX is able to reconstitute the four toxins, and we manage to reduce its scar size to facilitate their use. In addition, we prove the orthogonality of both inteins (iDnaE and iDnaX) through a toxin reconstitution assay, thus opening the possibility for complex set-ups based on these toxin–intein modules. This could be used to develop specific antimicrobial and other biotechnological applications.

Many synthetic biology developments aim at reprogramming bacteria to design and construct sophisticated new biological circuits, which already have multiple applications in biotechnology and medicine.^1−4^ However, if complexity of synthetic biological systems is ever increasing, it is accompanied by a parallel increase in potential parasite interactions with endogenous systems.^5^ The capacity of the components of a system to work independently, i.e., without interaction with other components, the orthogonality, is thus an essential prerequisite to construct robust synthetic biological systems.

One of the most urgent challenges in drug discovery is to find novel chemical or biological molecules that may act as antibacterial agents. Bacterial toxin–antitoxin (TA) systems are based on a two component module consisting of a toxic protein that inhibits cell growth or kills the bacteria, and an antitoxin molecule that modulates toxin activity.^6^ A few but important applications for TA systems as tools for biotechnology and molecular biology have been proposed.^7−10^ Type II TA systems involve, by definition, a toxic protein whose activity is inhibited by a proteic antitoxin. Toxins are small proteins with an efficient antibacterial activity for which spontaneous resistance is inexistent or extremely rare, because, although a toxin-resistant mutant has been generated,^7^ this was obtained after a laborious protocol. Consequently, toxins from the type II TA systems are potential drug targets^11^ and also candidates for the development of antimicrobials, as we previously showed.^12^

Inteins are dynamic protein sequences embedded within unrelated host proteins, from which they are excised in a maturation process called protein splicing. During the splicing process, the intein catalyzes its own excision, ligating the host protein flanks with a peptide bond and allowing the reconstitution of the mature protein, the extein (Figure S1). Inteins are considered to be disturbing extein activity, which is functionally compromised before protein splicing. Most of the inteins are located within one gene and catalyze their own splicing in *cis*, but naturally split inteins exist, in which the splicing domains are found in different genes (Figure S1). Inteins have been identified in the three domains of life, but they are particularly abundant in bacteria and archaea, where they generally interrupt genes that are essential for DNA replication and metabolism. Although recent advances in intein research may position them as mobile genetic elements and, as such, they might play a dynamic role in the evolution of species, their origin and possible role is still unclear.^13^

To further expand the applications of our antibacterial system,^12^ new toxin–intein units are needed. Among these potential applications is that of adapting it to other bacterial species, allowing us to potentially treat clinical cases caused by multiple bacteria. In addition, one of the practical solutions to reduce the development of resistance, a problem associated with antibacterial treatments, is the use of multiple killing systems. However, the multiplicity of combinations in a system based on toxin–intein units will only be scalable if these parts are orthogonal. In our setup, orthogonality should be operative for both parts: toxins and inteins. Apart from a recent work that showed a large set of split inteins and their orthogonality,^14^ there are very few studies addressing trans-splicing between inteins from different families.^15^ Here, we describe the construction of an artificially split intein named iDnaX.^16^ Using the constructions we previously developed with the wild type iDnaE split intein,^12^ we demonstrate that one can fully substitute the iDnaE split intein to reassemble the four toxins we previously tested. We further demonstrate that the parts of our four toxins are orthogonal—with both split inteins—i.e., they only produce functional toxin when partnered with their cognate half. Finally, we show that the four parts from our two inteins are orthogonal as well, as they only produce extein fusion when partnering with their cognate half.

Our results represent an advance in the availability of tools such as split toxins and inteins, and provide new parts for the development of smart antibiotics that could act simultaneously.

## Results and Discussion

**Figure 1:**
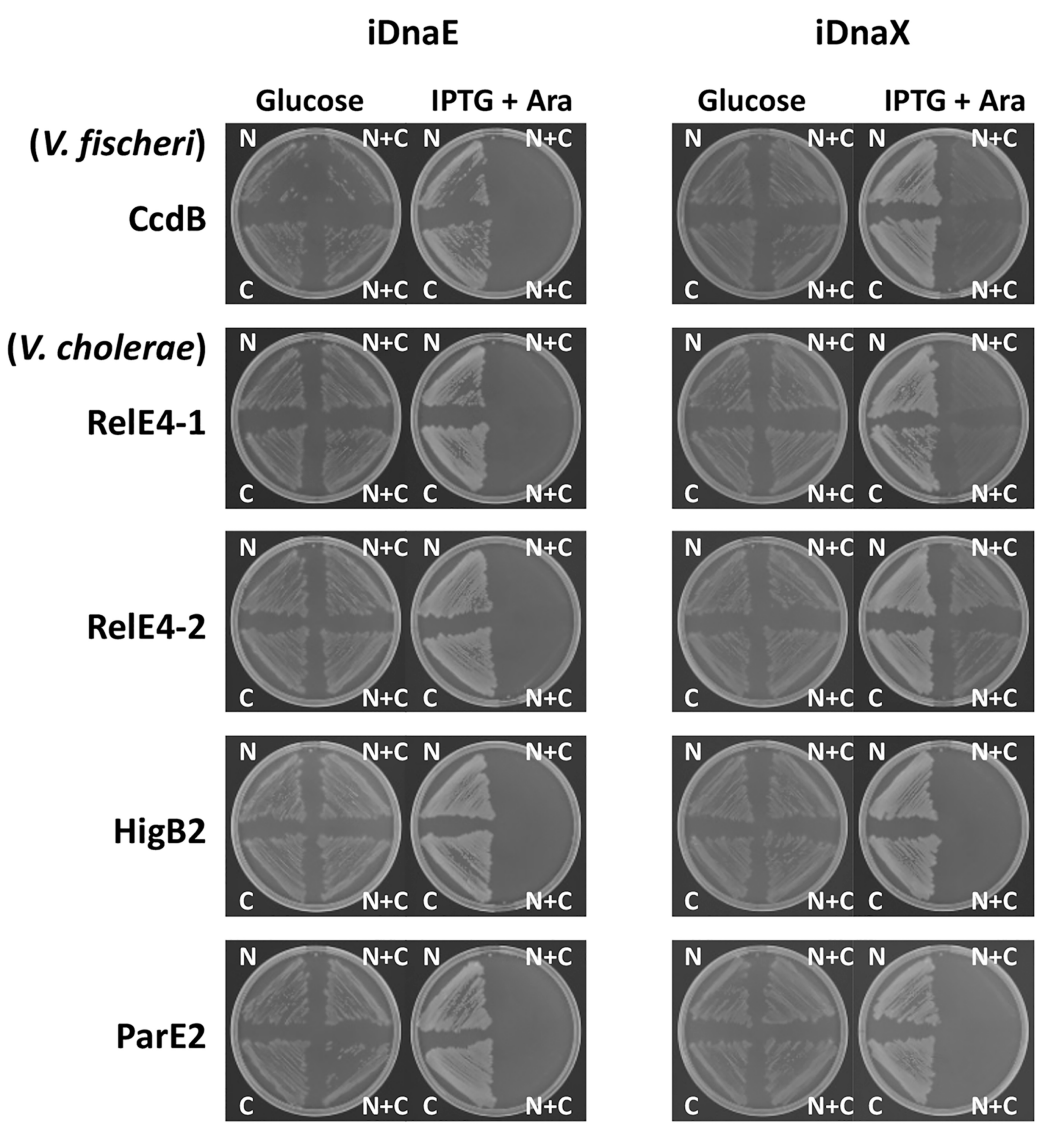
Intein–toxin activity assay *in vivo*. Growth on LB media with the antibiotics needed to maintain the two plasmids, in two different conditions: repressive (1% glucose) or inducible (1 mM IPTG and 0.2% arabinose). Each plate has three different plasmid combinations: “N” with N-terminal fusion plasmid, “C” with the C-terminal fusion plasmid and “N + C” with both plasmids. For “N” and “C”, the bacteria also carries the empty partner plasmidic vector to allow bacteria to grow on the same media.

We had selected the type II toxins based on the availability of their crystal structure, to avoid splitting the protein in a constrained domain, as intein excision leaves a scar of additional amino acids. The toxins we selected were CcdB from *Vibrio fischeri*, and RelE4, HigB2, and ParE2 from *Vibrio cholerae*. In our previous study,^12^ we split toxins into two fragments and fused them to the naturally split intein, DnaE from the cyanobacterium *Nostoc punctiforme*, named iDnaE (Figure S2). Here, we designed a new system with an intein that we artificially disrupted, iDnaX from the cyanobacterium *Synechocystis* sp. This intein had already been shown to function when split,^16^ avoiding the sequence for the homing endonuclease, but it was made in such a way that the scar left was of 6 specific amino acids (IDECHT). The first three (IDE) come from the N-terminal part and the last (CHT) are encoded into the C-terminal region being the natural sequence of one amino acid different (CHM). Here, we select the same split sequence based in the previous study^16^ and we reduce the remaining sequence to 3 amino acids (CHM) (Figure S3), keeping its natural sequence and demonstrating that the intein kept its functionality. Each toxin-split_intein (hereafter named S_intein) fusion part was cloned in a different compatible plasmid, as described for iDnaE.^12^ The reconstitution of functional toxins using iDnaX S_intein was found to be effective only when the two plasmids (N and C) were hosted together in the same cell (Figure 1). However, although the artificial iDnaX S_intein was able to perform protein splicing and reconstitution of all toxins tested, we observed a little background growth (Figure 1), which will be addressed below.

**Figure 2:**
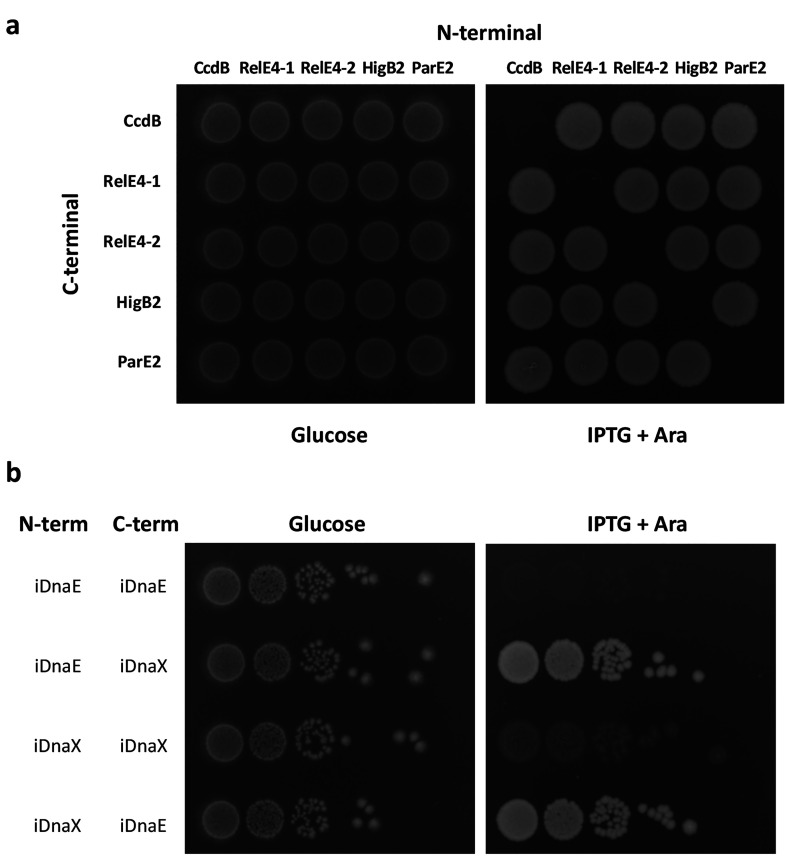
Orthogonality of toxins halves (a) and split inteins (b). *E. coli* spots on LB antibiotics and either 1% glucose or 1 mM IPTG and 0.2% arabinose, incubated at 37 °C overnight. Each spot corresponds to a bacteria harboring two plasmids: one carrying the N-terminal intein–toxin fusion listed in the first column, and the other plasmid with the C-terminal fusion as listed above the spots. (a) Orthogonality of toxin halves. In all tested fusions the intein is iDnaE, and the fused toxin is indicated for each combination of plasmids. (b) Orthogonality of intein halves. In all fusions the toxin is CcdB, and the fused intein half is indicated for each combination of plasmid. Spots are made from 10-fold serial dilutions.

Taking into account that the toxins used in this study belong to different families, we did not expect to get a toxic product after reconstitution by mixing their halves. However, it is possible that these artificial products could have collateral negative effects for the cell. In order to determine the orthogonality of our toxin system, we transformed *E. coli* MG1655 with all 25 pairwise combinations of toxin–intein fusion plasmids N and C for the same intein, iDnaE, and tested the growth in media containing either glucose, repressor, or IPTG and arabinose, the inducers of the expression for both parts (Figure 2a). Bacteria died only when the original toxin was reconstituted, but not in any other combination where the halves from different toxins were mixed. This orthogonality was also checked within the same toxin, RelE4, split in two different locations, where we also observed functional reconstitution only with the two cognate partners, i.e., split at the same location. We then can affirm that our toxin modules are orthogonal.

**Figure 3:**
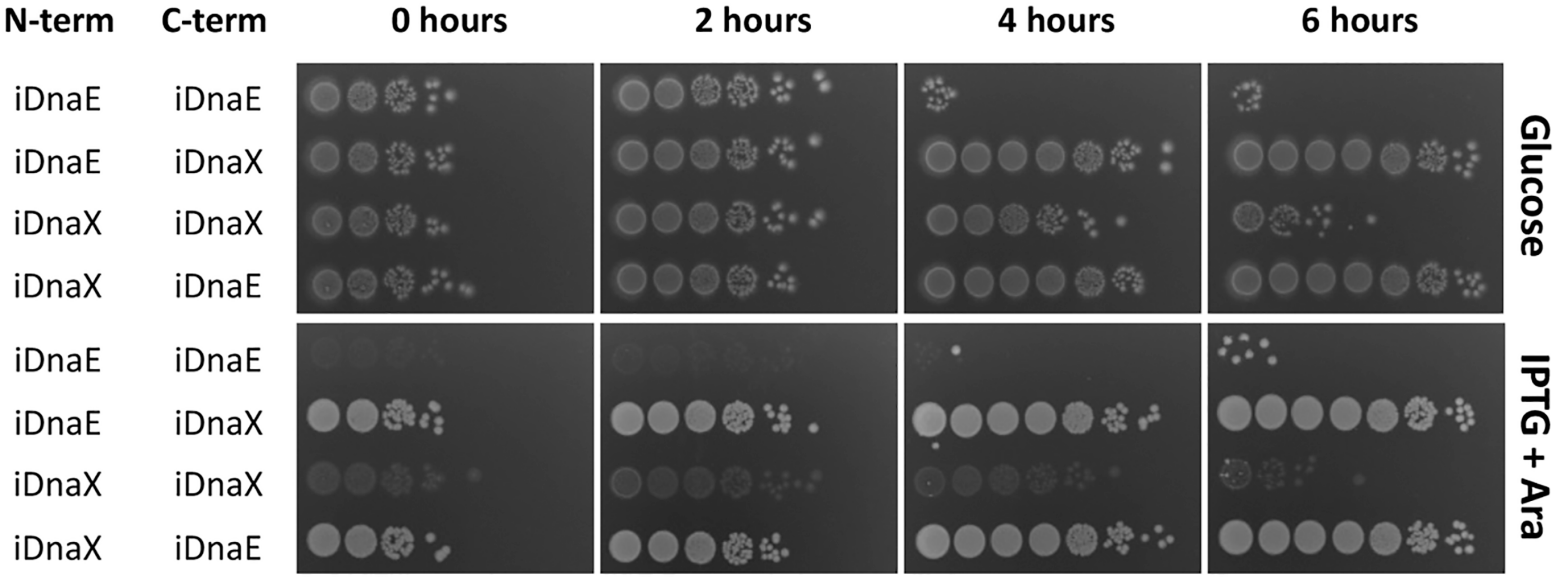
Intein splicing kinetics. Spots from the four different combinations of *ccdB*-iDnaE and *ccdB*-iDnaX on LB media containing the necessary antibiotics and either glucose (upper panel) or IPTG + arabinose (bottom panel). Overnight cultures in glucose and antibiotics were diluted 1/1000 in media containing IPTG and arabinose, incubated at 37 °C and spotted from 10-fold serial dilutions at different times, as indicated in the figure.

We also tested the orthogonality of our two split inteins. *Synechocystis* contains several inteins including iDnaX, and an iDnaE orthologous. iDnaE orthologous split inteins have been shown to have trans-splicing activity,^17,18^ and their sequences are highly conserved (Figure S4). Inside *Synechocystis*, as iDnaX is not naturally split, trans-splicing between iDnaX and iDnaE is not feasible. Here, our two split inteins, iDnaX from *Synechocystis* and iDnaE from *Nostoc*, belong to different families and only share the common features conserved among inteins, which are called blocks or motifs A, B, F, and G (Figure S4). We tested if intein splicing could occur by mixing the different halves of both inteins, using all different intein combinations for the reconstitution of the CcdB toxin (Figure 2b). The four combinations were cloned in *E. coli* MG1655 and tested in media with the appropriate inducers for protein expression (Figure 2). As observed for toxins, we found that bacteria die only when the reconstitution of the toxin is possible because of the splicing from cognate intein parts. Although a residual growth could be detected in the presence of IPTG and arabinose when toxin is reconstituted (Figure 2b), it disappeared after incubation in such conditions for 6 h (Figure 3). Thus, this demonstrates that both S_inteins, iDnaX and iDnaE, do not have intersplicing activity. Although iDnaX showed similarities with both orthologous iDnaE inteins (Figure S4), there are specific electrostatic and polar amino acids necessary for protein interaction in iDnaE intein^19^ that are not found in iDnaX sequence.

As mentioned above, when using iDnaX fusions with *V. cholerae* toxins RelE4 and ParE2, the killing is not as effective as with iDnaE, and leads to some background noise, i.e., a mat of very slow growing bacteria. We first tested if the residual sequence remaining in the toxin—which is different for both inteins after splicing (Figures S2 and S3)—could make a difference. In order to analyze that, these residual sequences were added into RelE4 toxin at split position 1 and 2, and after induction of its expression we did not detect any difference in toxicity (Figure S5). Thus, this *background noise* could be due to differences in the splicing rates between both inteins since iDnaE was described as an ultrafast splicing protein.^20^ This difference in splicing activity could reflect that efficient interactions between the two halves have not yet been optimized by selection, as iDnaX is not a naturally split intein. To evaluate the splicing dynamics of the two inteins, we performed time course experiments (Figure 3 and Figure S6). These results showed that whereas for CcdB and RelE4, bacteria containing iDnaE fusion are dead after 4 h of incubation with activator molecules, iDnaX fusion needs at least 4–5 h (Figure 3 and S6a). Note that iDnaX-RelE4 constructions showed higher variability and lower killing efficiency than the pairs iDnaE-RelE4 (Figure S6 and Table S3). On the other hand, HigB2 and ParE2 showed similar dynamics with both inteins but HigB2 presents a delay in terms of killing when compared with the other toxins (Figure S6 and Table S3). As we have already tested, this difference is not due to the residual sequence of both inteins (Figure S5) and we believe is a matter of splicing efficiency. These results, together with the fact that toxins are able to kill at low concentrations inside cells, make our system not only an easy platform to detect intein splicing, but also a highly sensitive one.

In this study, we demonstrated that, for two inteins and five toxins, the reconstitution of an effective toxin is only possible when the two halves of the cognate toxin–intein fusions are together in the same bacterium. The characterization of this system as orthogonal would help us to increase the complexity of the genetic circuits. Moreover, the different dynamics regarding intein splicing, toxin reconstitution and killing, give a flexible range of action to the system that could be useful to program sequential production of toxins. Toxin–intein combinations are a promising strategy for the development of smart antibiotics. Thus, orthogonality and sequential production could be useful to kill mutants escaping from a toxin–intein system using a second one. Altogether, our results show the vast potential of these combinations to move further in the design of this biotechnological application.

## Methods

### Cotransformation of *E. coli* MG1655 (or Top10)

*Escherichia coli* electrocompetent cells were generated as follows. Overnight cultures were setup in LB medium and incubated under shaking conditions at 37 °C. The next day, cultures were diluted 1:100 in fresh LB broth and grown at 37 °C, 200 r.p.m., to an optical density (OD_600nm_) of 0.8. Cells were cooled on ice for 30 min and washed three times with ice-cold 10% glycerol. Cells were finally resuspended in ice-cold 10% glycerol (1:1000 of the initial culture volume) and stored in aliquots at −80 °C. Plasmids were transformed in *Escherichia coli* by electroporation.

### Culture Conditions

After an overnight culture in LB media containing glucose to repress the expression of the fusions, we diluted the culture 1/1000 in media containing IPTG and arabinose and spotted 10 μL culture in plates also with IPTG and arabinose in order to induce the expression. Then culture could be induced for several hours (Figure 3 and Figure S6) in these conditions prior to spotting in LB solid media with the appropriate conditions. For the toxicity test with RelE4 and the mutants with residual sequences, the experiment was performed as described previously.^21^

### Plasmid Construction

Primers used in this work are listed in Table S1. Plasmid and strains used or constructed in this work are listed in Table S2. Plasmids for iDnaE intein fusions were constructed as described previously.^12^ To generate the N and C plasmids for DnaX-toxin fusions, the N- and C-terminal toxin regions were amplified with the primers F-toxin-*Eco*RI/R-toxin-DnaX and F-toxin-DnaX/R-toxin-XbaI, respectively. N- and C-terminal DnaX intein regions were amplified with the primers F-DnaX-toxin/R-DnaX-XbaI and F-DnaX-*Eco*RI/R-DnaX-toxin, respectively. We used chromosomal DNA from *V. cholerae* as a template for toxin genes *parE2*, *higB2*, and *relE4*, and *V. fischeri* DNA for *ccdB*. Intein amplification was done with chromosomal DNA from the cyanobacterium *Synechocystis sp.* PCC 6803. PCR products of N- and C-terminal regions were fused by Gibson assembly.^22^ Each toxin–intein fusion was then digested with *Eco*RI/XbaI (Thermo Fisher) and then cloned in *Eco*RI/XbaI digested pBAD43^23^ and pSU38^24^ plasmids, respectively.

For constructions of RelE4 toxin with the residual sequences CFN or CHM from iDnaE or iDnaX, respectively, we used the construction RelE4-pBAD43 that was previously done.^21^ Then, the additional sequences were added by performing PCR using this plasmid as a template and the following primers: F-RelE4-CFN-1/R-RelE4-CFN-1 or F-RelE4-CHM-1/R-RelE4-CHM-1, to add the CFN or CHM in the split site 1 from RelE-4, respectively; and F-RelE4-CFN-2/R-RelE4-CFN-2 or F-RelE4-CHM-2/R-RelE4-CHM-2, to add CFN or CHM in the split site 2, respectively. Gibson assembly was performed in order to ligate the PCR of the whole plasmid and then this reaction was used to transform *V. cholerae* (as in ref (25)), because this strain contained the antitoxin in their genome. Sanger sequencing was performed in order to check constructions. We then transformed *E. coli* MG1655 strain to do the assay (as explained before). Before carrying out the assay, we sequenced the plasmids again in order to be sure that we did not select mutations in the toxin.

## Supporting Information Available

Figure S1: Schematic illustration of protein splicing; Figure S2: Protein sequences for iDnaE intein from *Nostoc punctiforme*; Figure S3: Protein sequences for iDnaX intein from *Synechocystis* sp.; Figure S4: Amino acid sequence conservation by using Blast; Figure S5: Toxicity test of RelE4 toxin and its derivatives carrying the intein residual sequences; Figure S6: Growth test of *E. coli* MG1655 strain along time course after induction; Figure S7: Toxin–intein activity assay in different genetic backgrounds; Table S1: Bacterial strains and plasmids used in this study; Table S2: Primers used in this study; Table S3: Information summarizing the cell death along time in time course experiments (*n* = 3) (Figure S6) (PDF)
