# Supplementary material for: Increasing the Scalability of Toxin–Intein Orthogonal Combinations
Source: ACS Synth Biol. 2023 Jan 27;12(2):618–23. doi: 10.1021/acssynbio.2c00477 (PMC9942249; doi:10.1021/acssynbio.2c00477)
Supplement: Supplementary file 1 — sb2c00477_si_001.pdf [file sb2c00477_si_001.pdf]

Supporting Information

Increasing the scalability of toxin-intein orthogonal combinations

Rocío López-Igual[1,2*], Pedro Dorado-Morales[1] and Didier Mazel[1]

[1] Institut Pasteur, Université de Paris, Unité Plasticité du Génome Bactérien, et CNRS, UMR3525, 28 Rue du Dr Roux, F-75015 Paris, France

[2] Instituto de Bioquímica Vegetal y Fotosíntesis, CSIC and Universidad de Sevilla, Américo Vespucio 40, E-41092, Seville, Spain

*Corresponding author: Rocío López-Igual - Instituto de Bioquímica Vegetal y Fotosíntesis, CSIC and Universidad de Sevilla, Américo Vespucio 40, E-41092, Seville, Spain. Email address: mligual@us.es

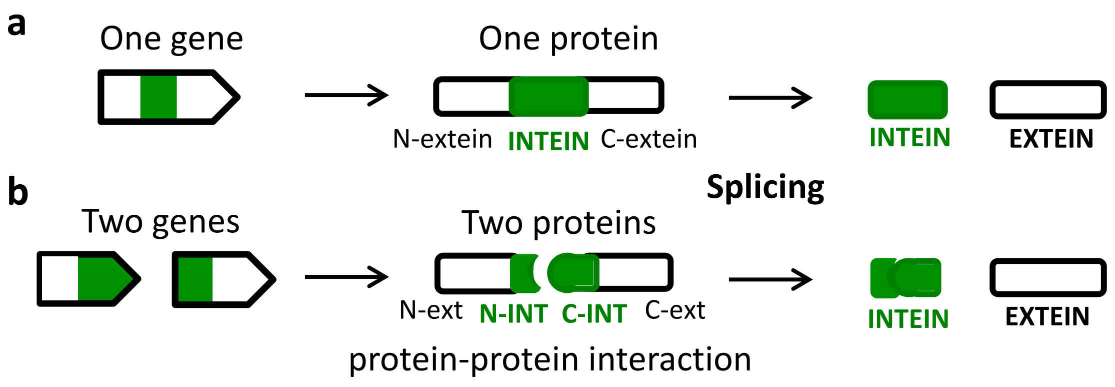

Figure S1. Schematic illustration of protein splicing of one-gene encoded intein (a) or split intein (b)

iDnaE intein **(two different proteins: Npun_F4872 and Npun_F5684)**
Extein boundary and Intein N-terminal sequence: FEQMLKFAEY/C
Intein C-terminal sequence and extein boundary: SN/CFNKSHSTAY

N-Intein Size (aa): 102
iDnaE-n N-terminal intein fragment (including -1 extein residue underlined)
YCLSYETEILTVEYGLLPIGKIVEKRIECTVYSVDNNGNIYTQPVAQWHDRGEQEVFEYCLEDG
SLIRATKDHKFMTVDGQMLPIDEIFERELDLMRVDNLPN
C-Intein Size (aa): 36
iDnaE-c C-terminal intein fragment (including +1 extein residue underlined)
MIKIATRKYLGKQNVYDIGVERDHNFALKNGFIASN**C**

iDnaE N-terminal fragment
CLSYETEILTVEYGLLPIGKIVEKRIECTVYSVDNNGNIYTQPVAQWHDRGEQEVFEYCLEDGS
LIRATKDHKFMTVDGQMLPIDEIFERELDLMRVDNLPN
iDnaE C-terminal fragment
MIKIATRKYLGKQNVYDIGVERDHNFALKNGFIASN/**CFN**

Figure S2. Protein sequences for iDnaE intein from *Nostoc punctiforme*. iDnaE is naturally split and two inteins fragments (102 and 36 aa, respectively) are encoded in different genes. The residual 3aa-sequence that remains in the final rejoined toxin (CFN) is highlighted in red and underlined.

iDnaX intein (one protein, Sll1360)
Intein Size (aa): 430
Extein boundary and Intein N-terminal sequence: CRYKVYVIDE/C
Intein C-terminal sequence and extein boundary: HN/CHMLSTAAFN

Intein aa Sequence
iDnaX intein (including -1 and +1 extein residues)

ECLTGDSQVLTRNGLMSIDNPQIKGREVLSYNETLQQWEYKKVLRWLDRGEKQTLSIKTK
NSTVRCTANHLIRTEQGWTRAENITPGMKILSPASVDVDNLSQSTALTASLGGLSGAINY
EAINTDKKNTTLSLSLKKQKPQDPFVNADVAKNLIFQHFCSAKEEKLKVSNPIGEDIPTK
KATDFGISEQKKLHQGQNRWEQKFSVLSTEPCLGMEVLTIPTHIADSPACDGPTAPSSQN
GWNIKRQDWDVCHPKYDSQPIKAMGKVPSAVKPVVPQTLLMFSAQSNLEVKENKFLRNGS
RISLKKEWLGGTWTTVPSLFPNLGVHQFSYTQRAFSRKKINLLLNGLPIEDIPPVQNPIA
EALTAKPITTQKWEQWPPASGYRTWKSIPSPQWHTNFEEVESVTKGQVEKVYDLEVEDNH
NFVANGLLVHNCHM

Figure S3. Protein sequences for iDnaX intein from *Synechocystis* sp. iDnaX is a non-naturally split intein. The two fragments created after artificial splitting (92 aa underlined in black and 44 aa underlined in brown-red, respectively) which were fused to different toxin genes. The residual 3 aa-sequence that remains in the final rejoined toxin (CHM) is highlighted in red. The sequence between the two underlined sequences of the intein encodes for a homing endonuclease (HEN).

a

```
Blast N-terminal intein domains:
                  *
DnaX Ssp   1    CLTGDSQVLT-RNGLMSIDNPQIKGREVLSYNETLQQWEY-KKVLRWLDRGEKQTLSIKT   58
                CL+ ++++LT   GL+ I     K  E   Y+         Y + V +W DRGE++
DnaE Npu   2    CLSYETEILTVEYGLLPIGKIVEKRIECTVYSVDNNGNIYTQPVAQWHDRGEQEVFEYCL   61
                      A

                    *  *
DnaX Ssp   59   KN-STVRCTANHLIRTEQG   76
                ++ S +R T +H    T  G
DnaE Npu   62   EDGSLIRATKDHKFMTVDG   80
                      B

Blast C-terminal intein domains:
                           ***
DnaX Ssp   19   EKVYDLEVEDNHNF-VANGLLVHNC   42
                + VYD+ VE +HNF + NG +   NC
DnaE Npu   13   QNVYDIGVERDHNFALKNGFIASNC   37
                      F             G
```

b

```
Blast N-terminal intein domains:
                                             *  *
DnaX Ssp   41   KKVLRWLDRGEKQTLSIKTKN-STVRCTANHLIRT   74
                + + +W DRGE++ L   + ++ S +R T++H   T
DnaE Ssp   43   QAIAQWHDRGEQEVLEYELEDGSVIRATSDHRFLT   77
                                            B

Blast C-terminal intein domains:
                               ***
DnaX Ssp     18   VEKVYDLEVEDNHNF-VANGLLVHNC   42
                  V++++D+ +   +HNF +ANG +   NC
DnaE Ssp     12   VQRIFDIGLPQDHNFLLANGAIAANC   37
                        F             G
```

c

```
Blast N-terminal intein domains:
                                                                  ** *      *
DnaE Ssp   1     YCLSFGTEILTVEYGPLPIGKIVSEEINCSVYSVDPEGRVYTQAIAQWHDRGEQEVLEYE   60
                 YCLS+ TEILTVEYG LPIGKIV + I C+VYSVD  G +YTQ +AQWHDRGEQEV EY
DnaE Npu   1     YCLSYETEILTVEYGLLPIGKIVEKRIECTVYSVDNNGNIYTQPVAQWHDRGEQEVFEYC   60
                 *
DnaE Ssp   61    LEDGSVIRATSDHRFLTTDYQLLAIEEIFARQLDLLTLENI   101
                 LEDGS+IRAT DH+F+T D Q+L I+EIF R+LDL+ ++N+
DnaE Npu   61    LEDGSLIRATKDHKFMTVDGQMLPIDEIFERELDLMRVDNL   101

Blast C-terminal intein domains:
                    *     *         *
DnaE Ssp   1     MVKVIGRRSLGVQRIFDIGLPQDHNFLLANGAIAANC   37
                 M+K+   R+ LG Q ++DIG+ +DHNF L NG IA+NC
DnaE Npu   1     MIKIATRKYLGKQNVYDIGVERDHNFALKNGFIASNC   37
```

Figure S4. Amino acid sequence conservation by using Blast. (a) Blast result of comparing iDnaX and iDnaE inteins, N-terminal or C-terminal domains as detailed. Blue lines and stars indicate the regions for inteins sequence blocks of motifs (A, B, F and G) which correspond to highly conserved domains as described in the InBase [1]. (b) Blast result of comparing iDnaE and iDnaX inteins from *Synechocystis* sp. (Ssp). As above, blue lines and stars indicate the regions for inteins sequence blocks that correspond to highly conserved domains. (c) Blast result of comparison between iDnaE inteins from *Synechocystis* sp. versus *Nostoc punctiforme* (Npu). Highlighted in red there are electrostatic residues, grey stars show non-polar residues; both type of residues are important for functional interactions and splicing [2].

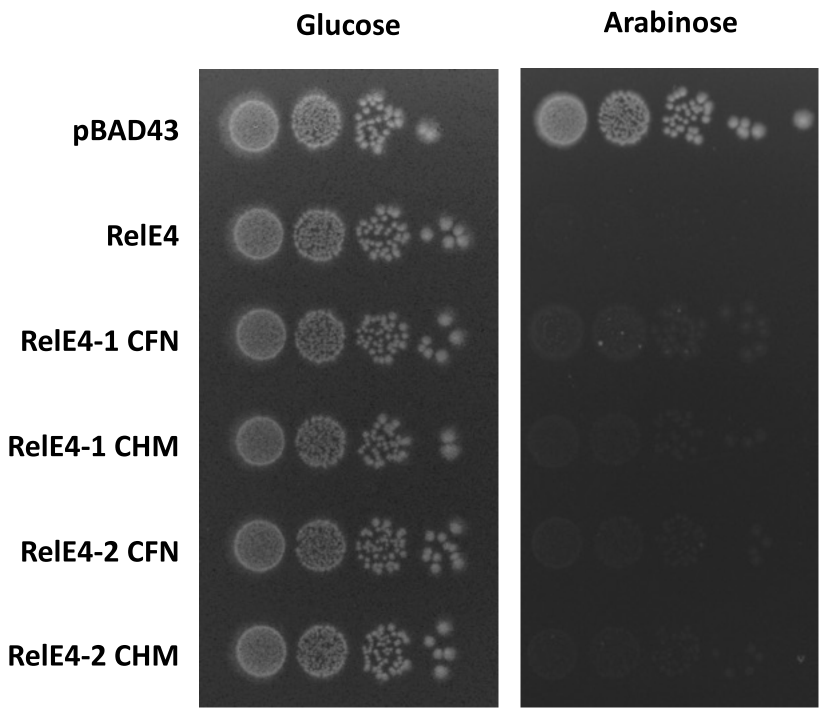

Figure S5. Toxicity test of RelE4 toxin and its derivatives carrying the intein residual sequences. LB solid media with spectinomycin and glucose (left panel) or arabinose (right panel). Spots correspond to MG1655 containing either the empty pBAD43, RelE4-pBAD43 or the mutants carrying the residual sequence "CFN" or "CHM" from iDnaE or iDnaX inteins, respectively, at split site 1 (RelE4-1) or split site 2 (RelE4-2), as indicated. Spots are made from 10-fol serial dilutions of the cultures.

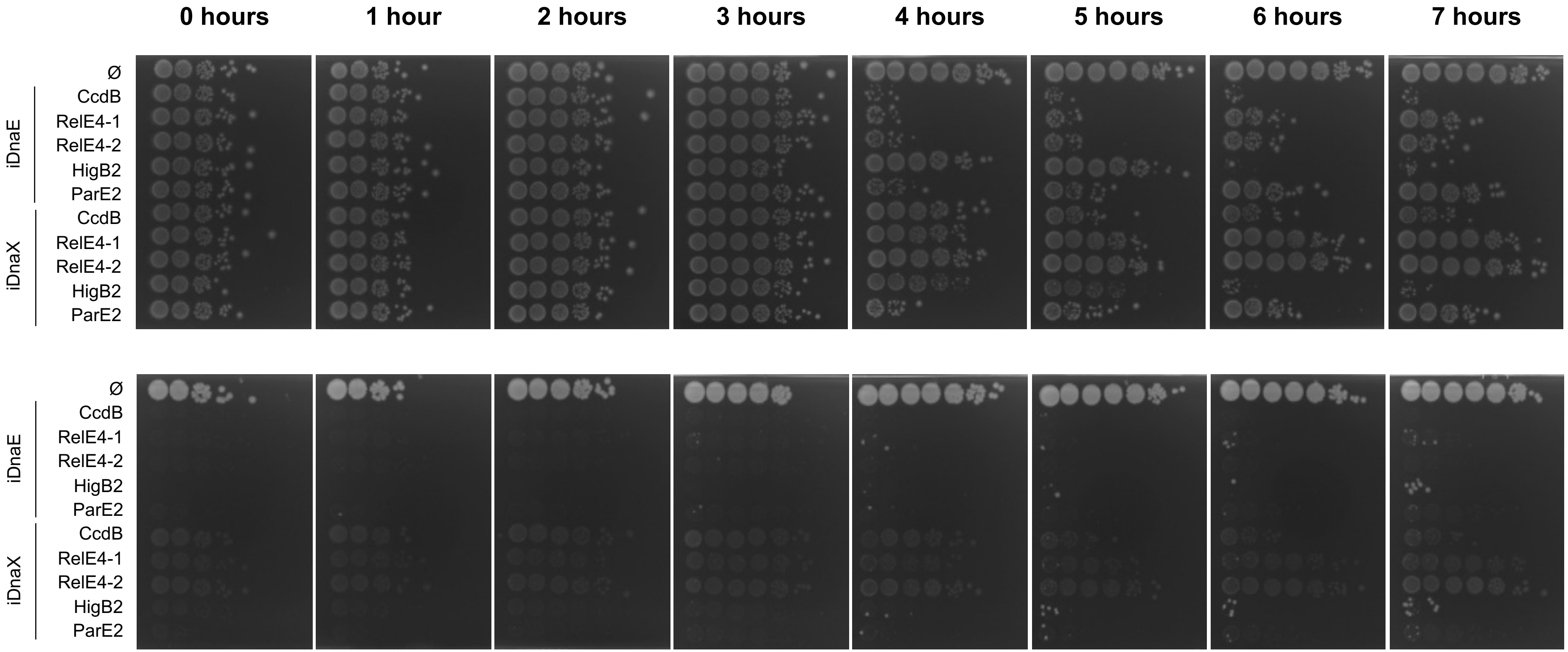

Figure S6. Growth test of *E. coli* MG1655 strain along time course after induction (IPTG and arabinose) or not (glucose) of all the toxin-intein fusions. Time in hours (h) indicate the time since incubation with IPTG and arabinose, before spotting. 10-fold serial dilutions of cultures spotted in LB with antibiotics containing either glucose (upper panels) or IPTG and arabinose (bottom panels) are shown for both inteins with all the toxins tested in this study. Strains containing empty plasmids (Ø) have been used as control for each condition.

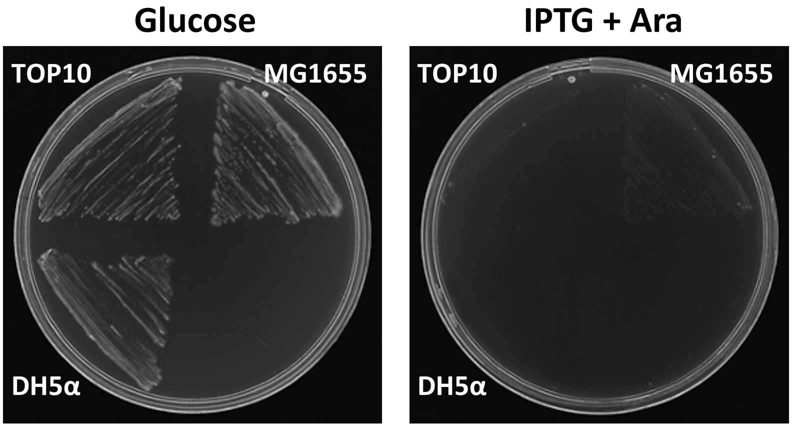

Figure S7. Toxin-intein activity assay in different genetic backgrounds. Growth on LB media with the antibiotics needed to maintain the two plasmids, in two different conditions: repressive (1% glucose) or inducible (1mM IPTG and 0,2% arabinose). Each plate contains three different *E. coli* strains (TOP10, MG1655 and DH5α) carrying the plasmids with the N and C-terminal *parE2*-iDnaX toxin-intein pairs.

Table S1: Bacterial Strains and Plasmids Used in This Study

Name	Relevant Features	Source/Reference
**Bacterial strains:**		
Escherichia coli		
MG1655	K-12 F- λ- *ilvG- rfb-50 rph-1*	Laboratory collection
DH5α	F⁻ *endA1 glnV44 thi-1 recA1 relA1 gyrA96 deoR nupG purB20* ϕ80d*lacZ*ΔM15 Δ(*lacZYA-argF*)U169, hsdR17($r_K^- m_K^+$), λ⁻	Laboratory collection
Top10	F- *mcrA* Δ(*mrr-hsd*RMS-*mcr*BC) Φ80*lac*ZΔM15 Δ *lac*X74 *rec*A1 *ara*D139 Δ(*ara-leu*)7697 *gal*U *gal*K *rps*L (StrR) *end*A1 *nup*G	ThermoFisher
Other strains		
Vibrio cholerae	*Vibrio cholerae* serotype O1 biotype El Tor strain N19691	(3)
Vibrio fischeri	WT	Laboratory collection
**Plasmids:**		
pBAD43	ori pSC101, Sp^R	(4)
pSU38	ori p15A, Kan^R	(5)
pCcdB-iDnaE N	pBAD43-ccdB/intein N-terminal fusion (EcoRI-XbaI)	(6)
pCcdB-iDnaE C	pSU38-ccdB/intein C-terminal fusion (EcoRI-XbaI)	(6)
pRelE4-iDnaE N-1	pBAD43-relE4/intein N-terminal-1 fusion (EcoRI-XbaI)	(6)
pRelE4-iDnaE C-1	pSU38-relE4/intein C-terminal-1 fusion (EcoRI-XbaI)	(6)
pRelE4-iDnaE N-2	pBAD43-relE4/intein N-terminal-2 fusion (EcoRI-XbaI)	(6)
pRelE4-iDnaE C-2	pSU38-RelE4/intein C-terminal-2 fusion (EcoRI-XbaI)	(6)
pHigB2-iDnaE N	pBAD43-higB2/intein N-terminal fusion (EcoRI-XbaI)	(6)
pHigB2-iDnaE C	pSU38-higB2/intein C-terminal fusion (EcoRI-XbaI)	(6)
pParE2-iDnaE N	pBAD43-parE2/intein N-terminal fusion (EcoRI-XbaI)	(6)
pParE2-iDnaE C	pSU38-parE2/intein C-terminal fusion (EcoRI-XbaI)	(6)
pCcdB-iDnaX N	pBAD43-ccdB/intein N-terminal fusion (EcoRI-XbaI)	This work
pCcdB-iDnaX C	pSU38-ccdB/intein C-terminal fusion (EcoRI-XbaI)	This work
pRelE4-iDnaX N-1	pBAD43-relE4/intein N-terminal-1 fusion (EcoRI-XbaI)	This work
pRelE4-iDnaX C-1	pSU38-relE4/intein C-terminal-1 fusion (EcoRI-XbaI)	This work

pRelE4-iDnaX N-2	pBAD43-relE4/intein N-terminal-2 fusion (EcoRI-XbaI)	This work
pRelE4-iDnaX C-2	pSU38-RelE4/intein C-terminal-2 fusion (EcoRI-XbaI)	This work
pHigB2-iDnaX N	pBAD43-higB2/intein N-terminal fusion (EcoRI-XbaI)	This work
pHigB2-iDnaX C	pSU38-higB2/intein C-terminal fusion (EcoRI-XbaI)	This work
pParE2-iDnaX N	pBAD43-parE2/intein N-terminal fusion (EcoRI-XbaI)	This work
pParE2-iDnaX C	pSU38-parE2/intein C-terminal fusion (EcoRI-XbaI)	This work
RelE4-pBAD43	pBAD43 containing RelE4 toxin from *V. cholerae*	(7)
pRelE4-CFN-1	RelE4-pBAD43 with CFN sequence in the split-site 1 for RelE4	This work
pRelE4-CHM-1	RelE4-pBAD43 with CHM sequence in the split-site 1 for RelE4	This work
pRelE4-CFN-2	RelE4-pBAD43 with CFN sequence in the split-site 2 for RelE4	This work
pRelE4-CHM-2	RelE4-pBAD43 with CHM sequence in the split-site 2 for RelE4	This work

Table S2: Primers used in this study.

Name	Sequence (5' → 3')
F-CcdB-EcoRI	GGAATTCATGTCTCAATTTACGCTATATAAAA
R-CcdB-XbaI	GCTCTAGAGCAAGCGTTAAATGCCAGTGATTA
F-iDnaE/dB	TAAGAAATGTTTAAGCTATGAAACGGAAATA
R-dB/iDnaE	CTTAAACATTTCTTATCAAGTAGTTCGATTGG
R-iDnaE-XbaI	GCTCTAGATCAATTCGGCAAATTATCAACC
F-iDnaE-EcoRI	GGAATTCGTAAGGAGGTAACATATGATCAAAATAGCCACACGTAAA
R-iDnaE/ccdB-2	CTTGGTGCATTGAAACAATTAGAAGCTATGA
F-CcdB-C/iDnaE 2	TTTCAATGCACCAAGTCACCTTTGTCCTAC
F-ParE2-EcoRI	GGAATTCAATGAAACCATTTAATCTTACCGTCGC
R-ParE2-XbaI	GCTCTAGATTATGCGCCGAATATTGGGTTCACATC
F-ParE2/iDnaE -1	TTTCAATATTGGTAAATCATGCGATGAAATCCGAG
R-ParE2/iDnaE -1	CTTAAACAGTCGGGATTTTCCGCTAAAAGCCA
F-iDnaE/ParE2-1	ATCCCGACTGTTTAAGCTATGAAACGGAAATA
R-iDnaE/ParE2-1	TTACCAATATTGAAACAATTAGAAGCTATGAAGC
F-HigB2-EcoRI	GGAATTCGATGAAAAGTGTATTTGTCGAATCAAC
R-HigB2 iDnaE-1	CTTAAACACTTTTCATCGAGAAAGTAATAG
F-HigB2/iDnaE-1	TTTCAATAGGCGTTTCTATTTGCTAACC
R-HigB2-XbaI	GCTCTAGATATCACGATTGCTCATTGCGCCACGCCTCC
F-iDnaE/HigB2-1	GATGAAAAGTGTTTAAGCTATGAAACGGAAATA
R-iDnaE/HigB2-1	GAAACGCCTATTGAAACAATTAGAAGCTATGAAGC
F-RelE4-EcoRI	GGAATTCGATGATTTTCTGGGAAGAAGCATCTCTCAATG
R-RelE4/iDnaE-1	CTTAAACAATCACGCTGAACACCGATTAG
F-RelE4/iDnaE-1	TTTCAATGGCATTAGAGGCAGATTGC
R-RelE4-XbaI	GCTCTAGATCAGTCGTTTGGAAATTTTTGTTTCTGATGTAG
F-iDnaE/RelE4-1	CAGCGTGATTGTTTAAGCTATGAAACGGAAATA
R-iDnaE/RelE4-1	CTAATGCCATTGAAACAATTAGAAGCTATGAAGC
R-RelE4/iDnaE-2	CTTAAACAATCAACCCAATACGAAACAATC
F-RelE4/iDnaE-2	TTTCAATGGTTCTAAAATTCGAATAATGCGTG
F-iDnaE/RelE4-2	GGGTTGATTGTTTAAGCTATGAAACGGAAATA
R-iDnaE/RelE4-2	TAGAACCATTGAAACAATTAGAAGCTATGAAGC
F-iDnaX-EcoRI	GGAATTCGTAAGGAGGTAACATATGCCGCAATGGCATACAAATTTCGAGGAAG
R-iDnaX-XbaI	GCTCTAGATCAAGGGGATAGTATCTTCATTCCGG
F-iDnaX-dB-N2	TAAGAAATGCTTAACGGGGGACTCACA
R-ccdB-iDnaX-N2	TAAGCATTTCTTATCAAGTAGTTCGATTGG
F-CcdB-C/iDnaX	CATATGGCACCAAGTCACCTTTGTCCTACG
R-iDnaX-dB-C	CTTGGTGCCATATGACAGTTATGGACTAG
F-HigB2/iDnaX	CATATGAGGCGTTTCTATTTGCTAACC
R-HigB2/iDnaX	TAAGCACTTTTCATCGAGAAAGTAATAG
F-iDnaX/HigB2	TGAAAAGTGCTTAACGGGGGACTCACAAG
R-iDnaX/HigB2	GAAACGCCTCATATGACAGTTATGGACTAG
F-ParE2/iDnaX	CATATGATTGGTAAATCATGCGATGAAATCCGAG
R-ParE2/iDnaX	TAAGCAGTCGGGATTTTCCGCTAAAAGCCA
F-iDnaX/ParE2	TCCCGACTGCTTAACGGGGGACTCACAAG
R-iDnaX/ParE2	TACCAATCATATGACAGTTATGGACTAG
F-RelE4/iDnaX-1	CATATGGGCATTAGAGGCAGATTGC
R-RelE4/iDnaX-1	TAAGCAATCACGCTGAACACCGATTAG

F-iDnaX/RelE4-1	GCGTGATTGCTTAACGGGGGACTCACAAG
R-iDnaX/RelE4-1	TAATGCCCATATGACAGTTATGGACTAG
F-RelE4/iDnaX-2	CATATGGGTTCTAAAATTCGAATAATGCGTG
R-RelE4/iDnaX-2	TAAGCAATCAACCCAATACGAAACAATC
F-iDnaX/RelE4-2	GGGTTGATTGCTTAACGGGGGACTCACAAG
R-iDnaX/RelE4-2	TAGAACCCATATGACAGTTATGGACTAG
F-RelE4-CFN 1	GATTGTTTCAATGGCATTAGAGGCAGATTGCTTATTATCC
R-RelE4-CFN 1	GCCATTGAAACAATCACGCTGAACACCGATTAG
F-RelE4-CHM 1	GATTGTCATATGGGCATTAGAGGCAGATTGCTTATTATCC
R-RelE4-CHM 1	GCCCATATGACAATCACGCTGAACACCGATTAG
F-RelE4-CFN 2	GATTGTTTCAATGGTTCTAAAATTCGAATAATGCG
R-RelE4-CFN 2	GGATTGAAACAATCAACCCAATACGAAACAATCATTG
F-RelE4-CHM 2	GATTGTCATATGGGTTCTAAAATTCGAATAATGCG
R-RelE4-CHM 2	GGCATATGACAATCAACCCAATACGAAACAATCATTG

Table S3: Information summarizing the cell death along time in time course experiments (n=3) (Fig. S6). Numbers in each toxin-intein combination represents the cell death detected in LB + glucose after the time in hours (h) incubated under inducible conditions, IPTG + arabinose.

Toxin / Intein	CcdB	RelE4-1	RelE4-2	HigB2	ParE2
iDnaE	4h	4h	4h	5-6h	4h
iDnaX	4-5h	4-5h	4-5h	4-6h	4h

High variability of cell death (1-4 Log)

Homogeneity of cell death (4-5 Log)

References :

1. Perler, F.B. (2000) InBase, the Intein Database. *Nucleic Acids Res*., **28**, 344-345.
2. Sorci, M., Dassa, B., Liu, H., Anand, G., Dutta, A. K., Pietrokovski, S., Belfort, M., Belfort, G. (2013). Oriented Covalent Immobilization of Antibodies for Measurement of Intermolecular Binding Forces between Zipper-like Contact Surfaces of Split Inteins. *Anal. Chem.* 85, 12, 6080–6088.
3. Heidelberg, J.F., et al. (2000) DNA sequence of both chromosomes of the cholera pathogen *Vibrio cholerae*. *Nature* 406(6795):477–483.
4. Guzman, L.M., Belin, D., Carson, M.J., Beckwith, J. (1995) Tight regulation, modulation, and high-level expression by vectors containing the arabinose P(BAD) promoter. *J Bacteriol* 177(14):4121–4130.
5. Bartolome, B., Jubete, Y., Martinez, E., de la Cruz, F. (1991) Construction and properties of a family of pACYC184-derived cloning vectors compatible with pBR322 and its derivatives. *Gene* 102(1):75–78.
6. ~~R.~~ López-Igual, R., ~~J.~~ Bernal-Bayard, J., ~~A.~~ Rodríguez-Patón, A., ~~J-M~~ Ghigo, J.M., and ~~D.~~ Mazel, D. (2019). Engineered toxin-intein antimicrobials can selectively target and kill antibiotic resistant bacteria in mixed populations. Nat. Biotech. Jul;37(7):755-760.
7. Iqbal, N., Guérout, A. M., Krin, E., Le Roux, F. & Mazel, D. Comprehensive functional analysis of the 18 Vibrio cholerae N16961 toxin-antitoxin systems substantiates their role in stabilizing the superintegron. J. Bacteriol. 197, 2150–2159 (2015).
